# An Amphipathic Alpha-Helix Domain from Poliovirus 2C Protein Tubulate Lipid Vesicles

**DOI:** 10.3390/v12121466

**Published:** 2020-12-18

**Authors:** Jobin Varkey, Jiantao Zhang, Junghyun Kim, Gincy George, Guijuan He, George Belov, Ralf Langen, Xiaofeng Wang

**Affiliations:** 1 Zilkha Neurogenetic Institute, University of Southern California, Los Angeles, CA 90033, USA; jvarkey@usc.edu (J.V.); jincyg38@gmail.com (G.G.); 2 School of Plant and Environmental Sciences, Virginia Tech, Blacksburg, VA 24061, USA; Jiantao.Zhang@som.umaryland.edu (J.Z.); guijuan@vt.edu (G.H.); 3 Virginia-Maryland College of Veterinary Medicine, University of Maryland, College Park, MD 20742, USA; jkim6615@umd.edu (J.K.); gbelov@umd.edu (G.B.)

**Keywords:** poliovirus 2C protein, positive-strand RNA virus, membrane remodeling, amphipathic alpha-helix, viral replication complex

## Abstract

Positive-strand RNA viruses universally remodel host intracellular membranes to form membrane-bound viral replication complexes, where viral offspring RNAs are synthesized. In the majority of cases, viral replication proteins are targeted to and play critical roles in the modulation of the designated organelle membranes. Many viral replication proteins do not have transmembrane domains, but contain single or multiple amphipathic alpha-helices. It has been conventionally recognized that these helices serve as an anchor for viral replication protein to be associated with membranes. We report here that a peptide representing the amphipathic α-helix at the N-terminus of the poliovirus 2C protein not only binds to liposomes, but also remodels spherical liposomes into tubules. The membrane remodeling ability of this amphipathic alpha-helix is similar to that recognized in other amphipathic alpha-helices from cellular proteins involved in membrane remodeling, such as BAR domain proteins. Mutations affecting the hydrophobic face of the amphipathic alpha-helix severely compromised membrane remodeling of vesicles with physiologically relevant phospholipid composition. These mutations also affected the ability of poliovirus to form plaques indicative of reduced viral replication, further underscoring the importance of membrane remodeling by the amphipathic alpha-helix in possible relation to the formation of viral replication complexes.

## 1. Introduction

Positive-strand RNA viruses [(+)RNA viruses] are the largest among all viral classes infecting eukaryotes, and include numerous important pathogens infecting humans, animals, and plants. A hallmark of (+)RNA virus replication is its dependence on host intracellular membranes to assemble functional viral replication complexes (VRCs) or replication organelles [1,2]. Different viruses replicate in specific organelle membranes, such as the ER, mitochondrion, chloroplast, vacuole, or peroxisome, among others [1,2]. Recent work using three-dimensional electron microscope tomography revealed critical information about the ultrastructure and organization of these membrane-bound VRCs of Flock house virus (FHV) at mitochondria [3,4], SARS coronavirus [5], Dengue virus [6], hepatitis virus C (HCV) [7], and beet black scotch virus [8] at the ER, tomato bushy stunt virus at peroxisome [9], and poliovirus at the interface of the ER and Golgi apparatus [10], among others. However, it is not well understood how viral replication proteins are targeted to specific organelles and how they interact with cellular membranes to remodel the membranes to form VRCs.

Multiple viral replication proteins have been reported to rearrange cellular membranes to form VRC-like structures, including brome mosaic virus (BMV) replication protein 1a [11], poliovirus 2C protein [12,13] or 3CD [14], and hepatitis C virus (HCV) non-structure protein 4B (NS4B) [15], among others. The 2C proteins are highly conserved among picornaviruses and play multiple roles in viral infection, including membrane remodeling, RNA binding, and packaging [16,17,18,19,20]. Poliovirus 2C protein has three structural domains: the central domain is an ATPase-helicase domain with the demonstrated ATPase activity [16], the C-terminus has a small cystine-rich region that binds zinc, and the N-terminus has a predicted amphipathic α-helix [17,18]. The expression of 2C or 2BC (the precursor of 2C) induces the reorganization of intracellular membranes and the formation of structures that morphologically resemble those found in PV-infected cells [12]. It was further shown that the ATPase is not required for the 2C-mediated membrane remodeling [12]. The 2C N-terminal fragment (amino acids 1–77) is sufficient to target soluble chloramphenicol acetyltransferase (CAT) to the ER membrane [19], and the expression of a fragment containing the first 122 amino acids of 2C also induces membrane rearrangements [20]. It has long been realized that poliovirus 2C protein has an amphipathic α-helix at its N-terminus (amino acids 19–36) that is required for membrane remodeling [17,18]. This N-terminal amphipathic α-helix is conserved among picornaviruses, and in addition, the helix in poliovirus 2C protein can be replaced by the counterpart from all tested enteroviruses, such as human enterovirus 71, coxsackie A virus 18, and human rhinovirus [18]. It was recently shown that 2C fused with a short Strep tag is targeted to lipid droplets (LDs). Deleting the N-terminal 39 amino acids (2C 40–329) or the amphipathic α-helix only (2C Δ17–38) resulted in a loss of association with LDs. Conversely, the first 38 amino acids with the helix are sufficient to be associated with LDs, suggesting that the helix plays an essential role in targeting 2C to its destination sites [21]. However, it has not been confirmed whether the predicted α-helix in poliovirus 2C protein behaves as a helix. If so, what are the consequences of the insertion of helix into the membranes, as an anchor for VRCs or other unrealized functions during the VRC formation?

It has been well-documented that cellular proteins (independent of viral infections) are intimately involved in remodeling membranes [22,23,24,25]. Various mechanisms have been suggested to explain how proteins induce membrane curvature [26]. These include scaffolding, in which the shape of the protein, for example, banana-shaped BAR domain proteins, defines the shape of the membrane [27], and wedging, in which asymmetric insertion of the protein or some region of the protein, like amphipathic α-helices, pushes the head groups apart, causing positive curvature [25,28]. The mechanism of wedging is similar to asymmetric insertion of a lipid with positive curvature that can push the lipid head groups apart due to its shape. The ability of amphipathic α-helices to induce curvature has been reported for many proteins, including α-synuclein, epsin, islet amyloid polypeptide, and α-helices present in many BAR domain proteins [25,26]. These amphipathic α-helices vary quite a bit in their length, charge, size of hydrophobic face, and composition of amino acids. Due to such variation, they differ in their affinities towards different phospholipid compositions, and hence, curvature-inducing abilities [25,26].

We report here that the synthesized peptide encompassing residues 19–36 of poliovirus 2C protein behaves like an amphipathic α-helix. We also show that the formation of amphipathic α-helix is accompanied by membrane modeling, similar to that reported for many non-viral proteins. In the absence of a transmembrane domain in the 2C protein, our results suggest that the association of the amphipathic α-helix of 2C protein with the membranes plays a critical role in viral replication.

## 2. Materials and Methods

### 2.1. Peptides

Lyophilized peptides were obtained from Peptide 2.0 at a purity greater than 95%. Protein concentration was estimated using the extinction coefficient of peptides at 280 nm based on the number of tryptophan molecules in the peptide. The peptides were dissolved in aqueous buffer.

### 2.2. Preparation of Phospholipid Vesicles

The following phospholipids were used to prepare different membrane compositions: 1-hexadecanoyl-2-(9Z-octadecenoyl)-sn-glycero-3-phosphate (POPA), 1-palmitoyl-2-oleoyl-SN-glycero-3-phosphocholine (POPC), 1-palmitoyl-2-oleoyl-sn-glycero-3-phosphoethanolamine (POPE), 1-palmitoyl-2-oleoyl-SN-glycero-3-[phospho-RAC-(1-glycerol)] (POPG), 1-palmitoyl-2-oleoyl-SN-glycero-3-phospho-L-serine (POPS), and L-α-phosphatidylinositol (PI) (Liver, Bovine). All phospholipids were purchased from Avanti Polar Lipids Inc. (Alabaster, AL, USA). Large unilamellar vesicles (LUVs) of different compositions were prepared by first preparing the required composition of phospholipids from stocks dissolved in chloroform, followed by drying it under nitrogen gas. The lipids were further kept under vacuum for complete removal of solvent for at least 4 h. The dried lipid was resuspended in an appropriate buffer and vortexed for 30 s. This was followed by treating the lipid solution to 10 cycles of freeze/thaw. The lipid mixture was finally passed through a mini extruder (20X) with a 1000 nm cutoff polycarbonate membrane (Avanti Polar Lipids Inc.) to generate large 1000 nm diameter vesicles.

### 2.3. Phospholipid Vesicle Clearance Assay

Peptides were tested for their ability to clear 1000 nm extruded phospholipid vesicles by measuring the change in light scattering as a function of time using a Jasco V-550 UV/Visible spectrophotometer. A wavelength of 500 nm with a slit width of 2 nm and medium response time was used to monitor the change in light scattering. Phospholipid vesicles were suspended in 20 mM of Hepes with a pH = 7.4 and 100 mM of NaCl at a final volume of 500 µL in a quartz cuvette. Large unilamellar vesicles (LUVs) composed of either 100% POPG, POPS/PI/POPC/POPE 15%/10%/50%/25% (termed as POPC/POPE 2:1 in the text and figures), or POPS/PI/POPC/POPE 15%/10%/37.5%/37.5% (termed as POPC/POPE 1:1 in the text and figures) were used.

### 2.4. Dye Leakage Assay

Leakage assay was modified from a previous method [25]. Large unilamellar vesicles (LUVs) composed of either 100% POPG, POPS/PI/POPC/POPE 15%/10%/50%/25% (termed as POPC/POPE 2:1 in the text and figures) or POPS/PI/POPC/POPE 15%/10%/37.5%/37.5% (termed as POPC/POPE 1:1 in the text and figures) were prepared by resuspending dried lipid in a mixture of 9 mM of ANTS (8-aminonaphthalene-1,3,6-trisulfonic acid, disodium salt) and 25 mM of DPX [p-xylene-bis (pyridinium bromide)] (Invitrogen, Thermo Fisher Scientific). This solution was then treated with 10 cycles of freeze/thaw. To prepare large 1000 nm diameter vesicles, the mixture was passed through a 1000 nm cutoff polycarbonate membrane (Avanti Polar Lipids Inc.) using a mini extruder. The unencapsulated dye was separated from vesicles by gel filtration using a PD-10 column (GE). One hundred percent leakage was attained using a final concentration of 0.1% Triton-X 100. All data were normalized to 100% leakage. Percent leakage was calculated using the formula y2−y1/y3−y1×100, where y1 is the fluorescence intensity of intact vesicles, y2 is the fluorescence intensity after the addition of peptide, and y3 is the fluorescence intensity after the addition of Triton-X. Fluorescence measurements were recorded using a JASCO fluorometer (FP-6500), setting excitation and emission at 380 nm and 520 nm with slits of 5 nm and 20 nm, respectively.

### 2.5. Transmission Electron Microscopy

Negatively stained specimens were prepared by floating carbon-coated formvar films mounted on copper grids (Electron Microscopy Sciences) on 10 µL droplets of samples for 5 min, followed by blotting the excess liquid and staining with 1% uranyl acetate. A JEOL 1400 transmission electron microscope operated at 100 kV was used for imaging. Large unilamellar vesicles (LUVs p-xylene-bis (pyridinium bromide)) composed of either 100% POPG, POPS/PI/POPC/POPE 15%/10%/50%/25% (termed as POPC/POPE 2:1 in the text and figures), or POPS/PI/POPC/POPE 15%/10%/37.5%/37.5% (termed as POPC/POPE 1:1 in the text and figures) were used.

### 2.6. Circular Dichroism (CD)

CD spectra were collected using a Jasco J-810 spectropolarimeter with a 1 mm quartz cell at room temperature. The following parameters were used for measurement: a scan rate of 50 nm/minute, step resolution of 0.5 nm, 0.1 nm time response, and a bandwidth of 1 nm. Protein concentration was estimated using the extinction coefficient of peptides at 280 nm on the basis of tryptophan molecules in the peptides. The final spectra were obtained by measuring appropriate blanks under similar conditions and subtracting them from the sample spectra. A 10 mM sodium phosphate (pH = 7.4) buffer was used for all CD studies. Large unilamellar vesicles (LUVs) composed of either 100% POPG, POPS/PI/POPC/POPE 15%/10%/50%/25% (termed as POPC/POPE 2:1 in the text and figures), or POPS/PI/POPC/POPE 15%/10%/37.5%/37.5% (termed as POPC/POPE 1:1 in the text and figures) were used.

### 2.7. Plaque Assay for Testing RNA Infectivity of wt and L3A Mutant Polioviruses

To generate L3A mutant poliovirus (PV-L3A), the three mutations were induced into the pXpA-SH, a plasmid containing full-length cDNA of poliovirus (PV-wt), via mutagenesis. For the RNA infectivity assay, full-length polio RNA of PV-wt or PV-L3A was synthesized with Ambion T7 RNA MEGA Script transcription kit (Thermo Fisher Scientific) using corresponding plasmid as the template [29]. HeLa cells grown on six well plates were transfected with serial dilutions of the transfection mix prepared using transIT mRNA transfection reagent (Mirus) and polio RNA according to the manufacturer’s protocol. Six hours post-transfection, the cells were overlaid with DMEM growth medium containing 2% FBS solidified with 1% agarose. To visualize plaques, the monolayer was stained with crystal violet at two days post RNA transfection.

## 3. Results

### 3.1. A Helix Domain from the Poliovirus 2C Protein Displays Amphipathic Propensity

**Figure 1:**
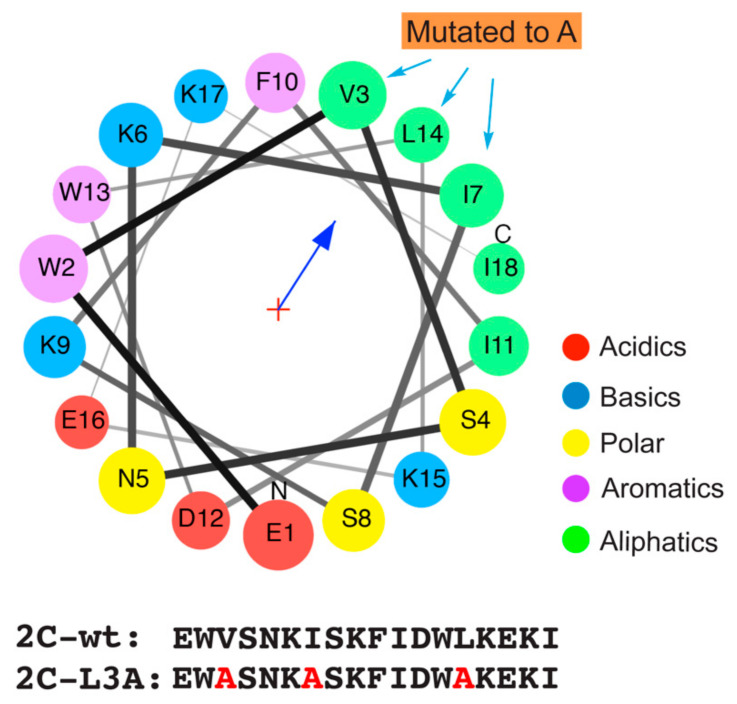
Helical wheel projection of the α-helix from poliovirus 2C protein in the N-terminal region. 2C-wt: amino acids 19–36 of poliovirus 2C; 2C-L3A: Amino acids at position 3, 7, and 14 were mutated to alanine. Red, acidic amino acid; blue, basic amino acid; yellow, polar amino acid; purple, aromatic amino acid; green, aliphatic amino acid.

Previous studies have shown the requirement of the N-terminal region within the poliovirus 2C protein for its membrane association [17,18,21]. In this study, we evaluated the role of an 18 amino acid peptide (2C-wt) from the N-terminal region of the poliovirus 2C protein (amino acids 19–36) for its ability to interact with membranes (Figure 1). The helical wheel projection showed that the peptide possessed the ability to form an amphipathic α-helix with a hydrophobicity (H) of 0.440 and a hydrophobic moment (μH) at 0.526. Hydrophobic moment is a measure of the amphipathicity perpendicular to the axis of any periodic peptide structure, like an α-helix or a β-sheet. It can be calculated for a peptide using the specific hydrophobicity values associated with each amino acid. The hydrophobicity values are used to weight vectors for each amino acid residue as they are positioned around the helix. The hydrophobic moment is the summation of the vectors. A mutated peptide (2C-L3A) was designed to study the disruption of the hydrophobic face of the putative amphipathic peptide. Amino acids at positions 3 (V3), 7 (I7), and 14 (L14) were mutated to alanine, thereby affecting the hydrophobicity (H) of the peptide at 0.229 and a μH at 0.330.

### 3.2. The α-Helix Domain Transforms Large Vesicles into Small Structural Entities

**Figure 2:**
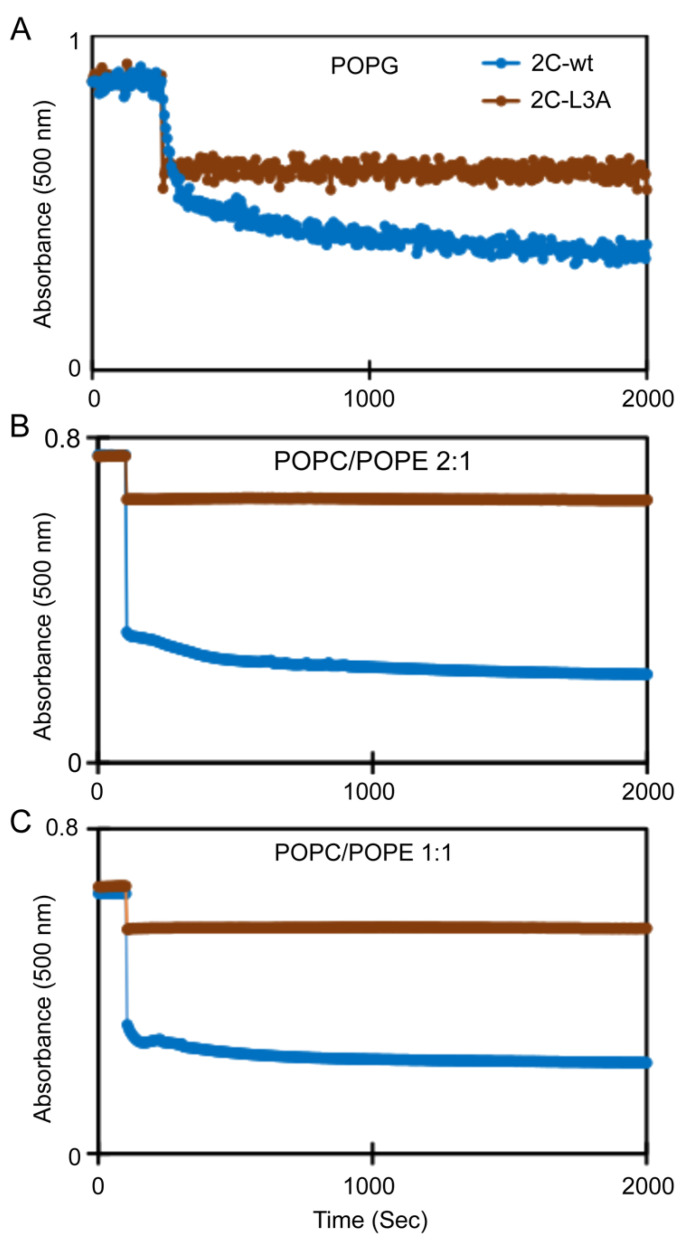
Clearance of lipid vesicles by 2C peptides. (**A**) POPG vesicles; (**B**) POPC/POPE 2:1 vesicles; (**C**) POPC/POPE 1:1 vesicles. The molar ratio of protein/lipid was 1:10.

The 2C protein has been reported to affect membrane architecture. To test whether the amphipathic α-helix is sufficient to cause membrane restructuring, we monitored the ability of both 2C-wt and 2C-L3A to transform large unilamellar vesicles using a vesicle clearance assay that has been previously employed in studies reporting membrane remodeling by amphipathic α-helices [25]. In this assay, a decrease in apparent absorbance was correlated with a decreased light scatter that was due to the remodeling of large vesicles into smaller structures, such as small vesicles or tubules. Both peptides transformed the large POPG vesicles into smaller structures, although 2C-wt displayed a higher clearance than 2C-L3A, as observed by a decrease in apparent absorbance due to scatter (Figure 2A). Since 100% POPG vesicles are highly negatively charged, we used vesicles that were more physiologically relevant. We used two lipid compositions that were representative of the endoplasmic reticulum membrane [30]: one with a 2:1 POPC/POPE molar ratio and another with a 1:1 POPC/POPE molar ratio, since viral replication is known to affect the lipid metabolism, resulting in an increased production of phosphatidylcholine lipids in certain cases [31]. The 2C-wt peptide still displayed a significant transformation of large lipid vesicles with both POPC/POPE 2:1 and POPC/POPE 1:1 lipid vesicles (Figure 2B,C). A slightly higher vesicle clearance was observed with POPC/POPE 2:1 vesicles when compared to POPC/POPE 1:1 lipid vesicles (Figure 2B,C). The vesicle clearing ability of 2C-L3A seemed to be highly compromised in the presence of both POPC/POPE 2:1 and POPC/POPE 1:1 lipid vesicles (Figure 2B,C).

### 3.3. An Increase in Helical Conformation Is Associated with Membrane Binding and Remodeling

**Figure 3:**
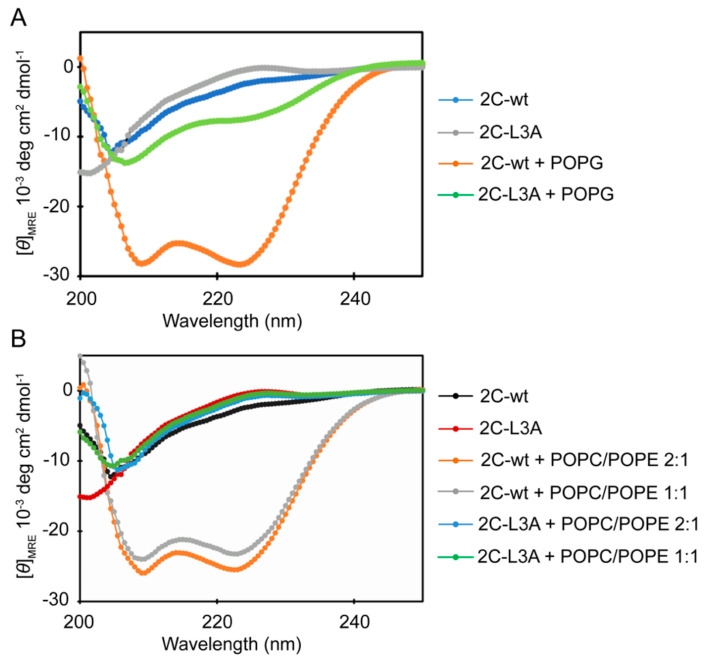
Circular dichroism spectra of 2C-wt and 2C-L3A with and without liposomes. (**A**) CD spectrum of 2C-wt and 2C-L3A in the presence of negatively charged POPG vesicles; 2C-L3A has drastically reduced helicity compared to 2C-wt. (**B**) CD spectrum of 2C-wt and 2C-L3A in the presence of a different ratio of POPC/POPE; 2C-L3A displays reduced helicity compared to 2C-wt. The peptide concentration was 100 μM and the lipid concentration was 500 μM.

We used circular dichroism to observe any conformational changes associated with the peptide during vesicle clearance. Minima at 208 nm and 222 nm indicated the presence of α-helical conformation in a protein. Both peptides, 2C-wt and 2C-L3A, displayed spectra characteristic of α-helical structure in the presence of 100% negatively charged POPG vesicles (Figure 3A). However, 2C-L3A had a highly reduced helicity when compared to the 2C-wt peptide. This could be either due to a reduced number of bound peptides to the lipid vesicles in the case of 2C-L3A, or peptides bound to the lipid vesicle with a partial helical structure. This difference is magnified in the presence of POPC/POPE 2:1 and POPC/POPE 1:1 vesicles (Figure 3B); 2C-L3A shows a complete loss of conformational change with both POPC/POPE 2:1 and POPC/POPE 1:1 vesicles. The three mutations introduced to affect the hydrophobicity of the 2C helical domain seemed to significantly affect the membrane binding property of this domain. Similar to the vesicle clearance data, we observed a slightly higher helicity with POPC/POPE 2:1 vesicles compared to POPC/POPE 1:1 vesicles (Figure 2B, compare orange and grey lines).

### 3.4. Large Unilamellar Vesicles Are Remodeled into Tubular Structures by the Helix Domain from 2C Protein

**Figure 4:**
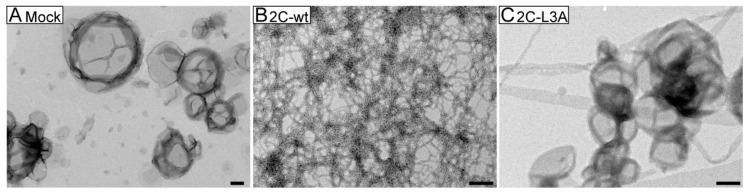
Remodeling of negatively charged vesicles by 2C peptides; 1000 nm negatively charged POPG vesicles were not treated (Mock; (**A**)) or treated with 2C-wt (**B**) or 2C-L3A (**C**) peptides. The peptide concentration was 50 μM and the lipid concentration was 500 μM. Scale bar = 200 nm.

**Figure 5:**
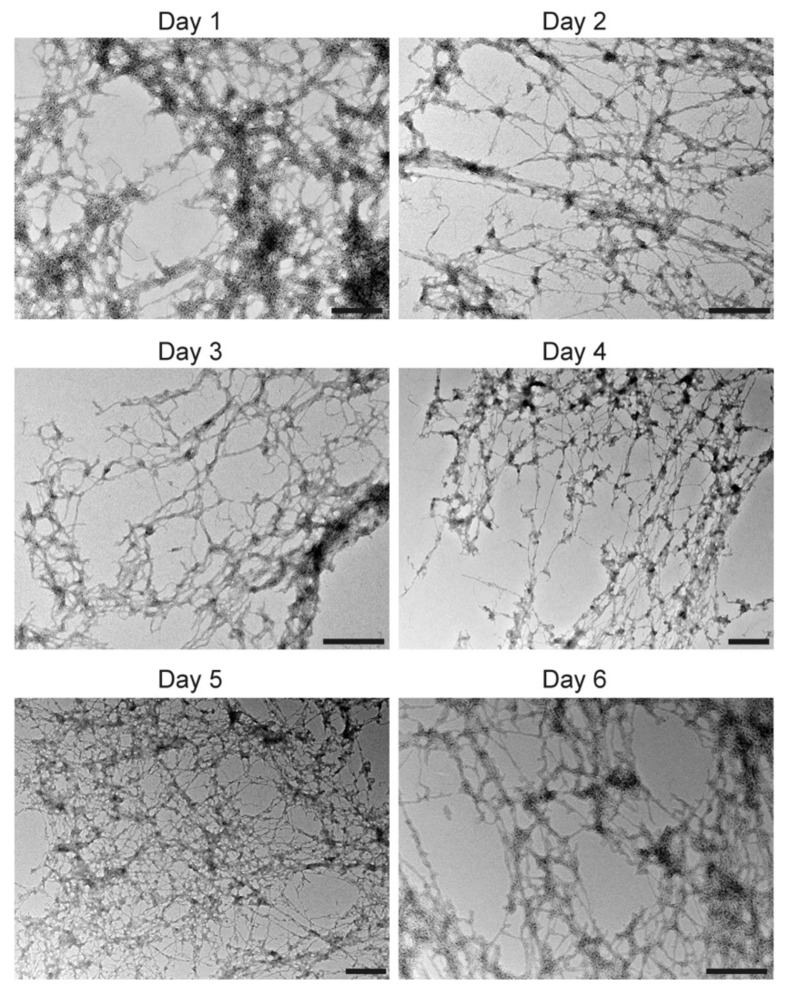
Stability of membrane tubes formed by the peptide 2C-wt; 2C-wt peptides (50 μM) were incubated with 1000 nm POPG vesicles (500 μM). The tubes were imaged for six days using transmission electron microscopy. No change in tube morphology was observed until day six. Scale bar = 500 nm.

In order to examine the morphological changes associated with lipid vesicles, we visualized them using transmission electron microscopy. First, we used POPG vesicles with which maximum vesicle clearance was observed (Figure 3). Both 2C-wt and 2C-L3A displayed the ability to remodel larger vesicles (Figure 4A) into tubular membranes (Figure 4B,C). However, similar to vesicle clearance data and structural data, a drastic reduction in membrane remodeling was observed with 2C-L3A (Figure 4C). The tubes formed by the 2C-wt peptide were stable even up to six days, suggesting a stable association of the peptide to the lipid (Figure 5). A similar behavior has been previously observed with other membrane remodeling amphipathic α-helical proteins, such as α-synuclein [25].

**Figure 6:**
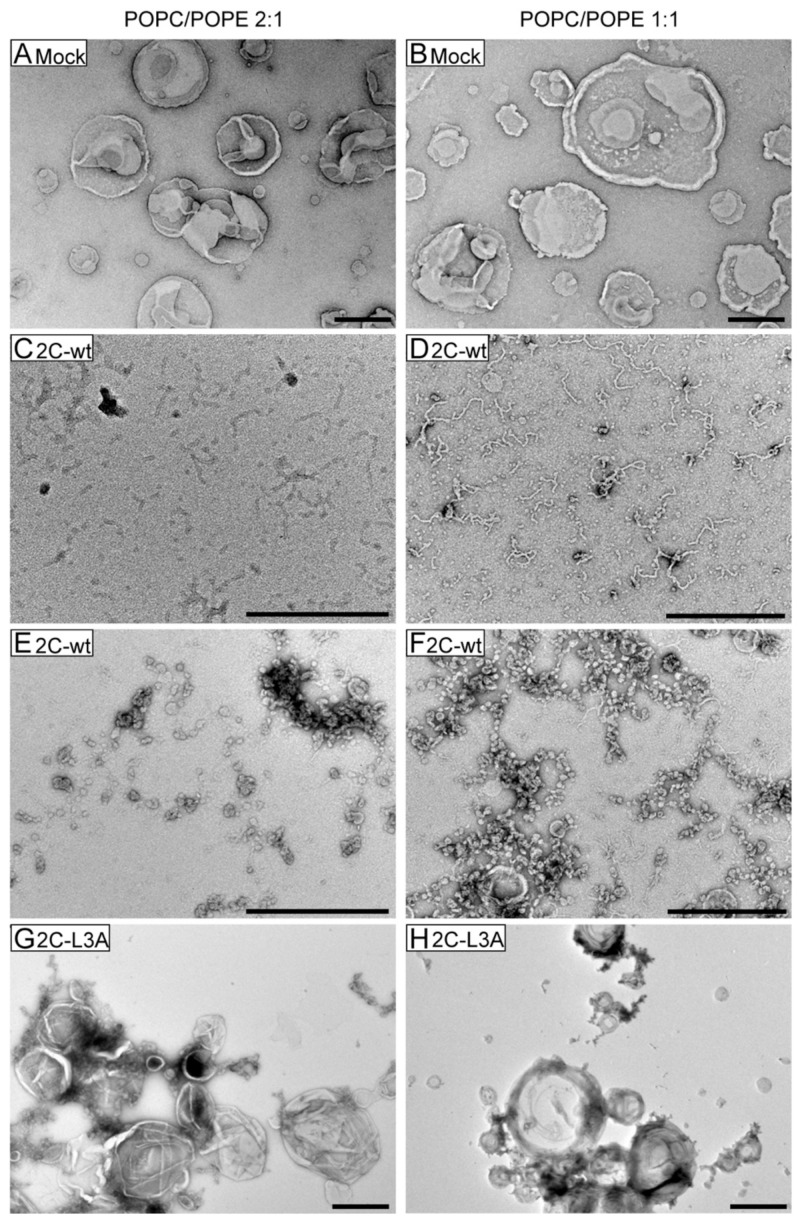
2C-wt but not 2C-L3A remodels vesicles with physiologically relevant lipid compositions. Peptide 2C-wt or 2C-L3A (100 μM) was incubated with 500 μM of POPC/POPE 2:1 (left column) or POPC/POPE 1:1 (right column) vesicles. Lipid vesicles of POPC/POPE 2:1 (**A**) or POPC/POPE 1:1 (**B**) in the absence of peptides. Small tubes (**C**,**D**) or small vesicular structures (**E**,**F**) were formed when POPC/POPE 2:1 (**C**,**E**) or POPC/POPE 1:1 (**D**,**F**) vesicles were incubated with 2C-wt. No structural changes were observed for POPC/POPE 2:1 (**G**) or POPC/POPE 1:1 (**H**) vesicles in the presence of 2C-L3A. Scale bar = 500 nm.

Membrane remodeling was also observed with POPC/POPE 2:1 and POPC/POPE 1:1 vesicles (Figure 6). However, here we observed the remodeling of vesicles into two types of smaller tubules (Figure 6C–F), as opposed to the very long membrane tubes that were observed with POPG vesicles (Figure 4B). The first group was smaller tubes, as shown in Figure 6C for POPC/POPE 2:1 and Figure 6D for POPC/POPE 1:1. The second group was smaller vesicular structures, as demonstrated in Figure 6E for POPC/POPE 2:1 and Figure 6F for POPC/POPE 1:1. No membrane remodeling was observed with POPC/POPE 2:1 or POPC/POPE 1:1 vesicles in the presence of the 2C-L3A peptide (Figure 6G,H).

### 3.5. Tubulation Is Accompanied by Vesicle Leakage

**Figure 7:**
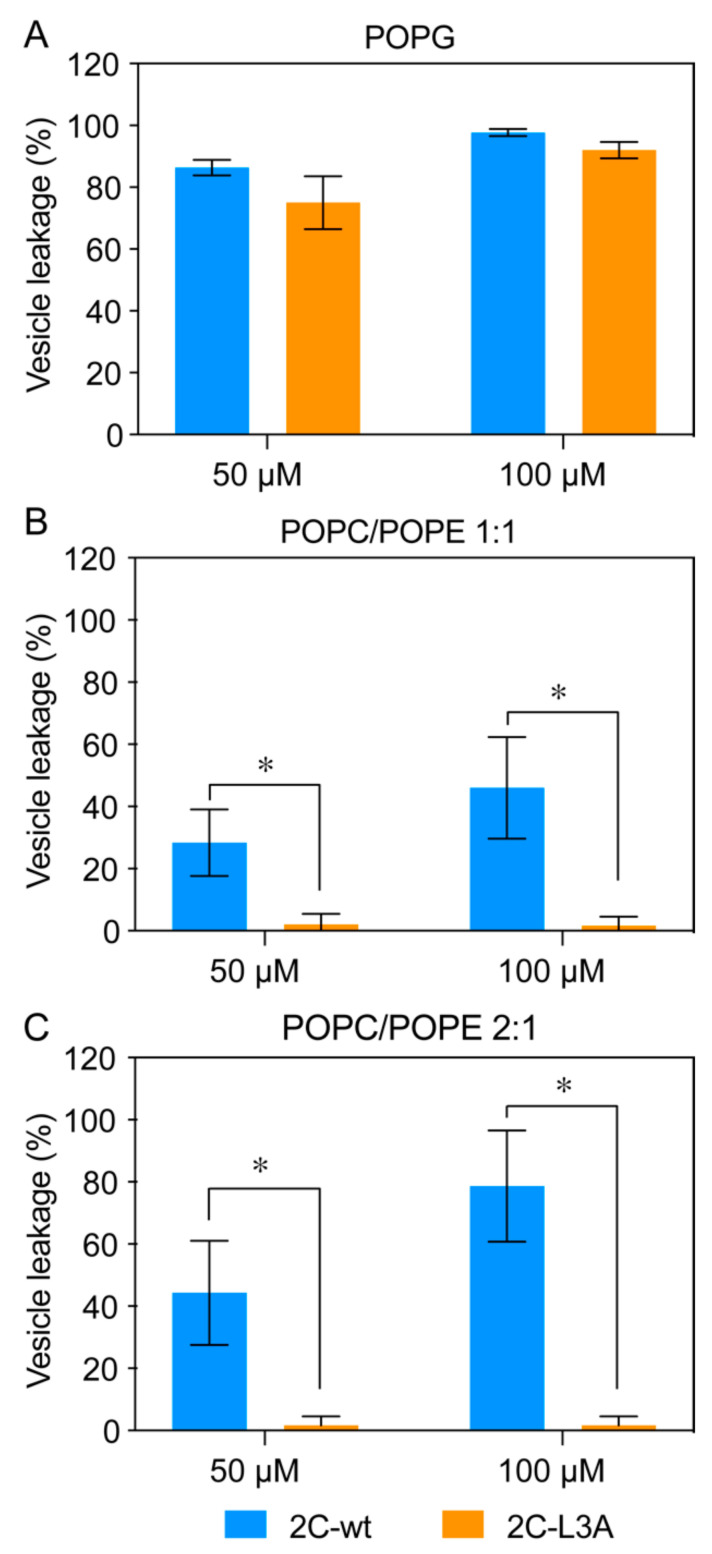
Vesicle leakage induced by 2C peptides. A significantly higher amount of leakage is observed with 2C-wt compared to 2C-L3A. POPG (**A**), POPC/POPE 1:1 (**B**), or POPC/POPE 2:1 (**C**) vesicles were incubated with 2C-wt (blue) or 2C-L3A (orange) peptides. The concentrations of peptides were tested at 50 and 100 μM. Lipid vesicles were used at 500 μM concentration. Data are represented as the mean ± SE of three independent experiments. *, *p* < 0.05 (paired Student’s *t*-test).

We inferred that a robust membrane remodeling that results in a significant change of membrane architecture would be associated with membrane disruption. To monitor this, we employed a vesicle leakage assay. The intact vesicles contained both ANTS, a fluorophore, and DPX, a quencher of fluorescence. Any disruption of liposomes that resulted in the leakage of ANTS or DPX would separate the fluorophore from the quencher and cause an increase in the fluorescence, therefore, an increase in fluorescence was correlated with an increase in leakage. Our data showed a significant leakage by both 2C-wt and 2C-L3A peptides with highly negatively charged POPG vesicles when 50 or 100 μM peptides were used (Figure 7A). Although 2C-L3A displayed a reduced leakage compared to 2C-wt, the difference was not statistically significant. However, a significant difference (*p* < 0.05) was observed between 2C-wt and 2C-L3A peptides when they were incubated with POPC/POPE 2:1 (Figure 7B) or POPC/POPE 1:1 (Figure 7C) vesicles in that 2C-L3A displayed a significantly reduced leakage compared to 2C-wt. It is worth noting that 2C-L3A peptides did not show any noticeable leakage with POPC/POPE 2:1 or POPC/POPE 1:1 vesicles (Figure 7B,C). A higher percentage of leakage was observed with POPC/POPE 2:1 vesicles compared to POPC/POPE 1:1 vesicles, which was in accordance with vesicle clearance and structural data using circular dichroism.

### 3.6. Mutations Reducing Helicity of the Amphipathic α-Helix of 2C Protein Block Poliovirus Infection

**Figure 8:**
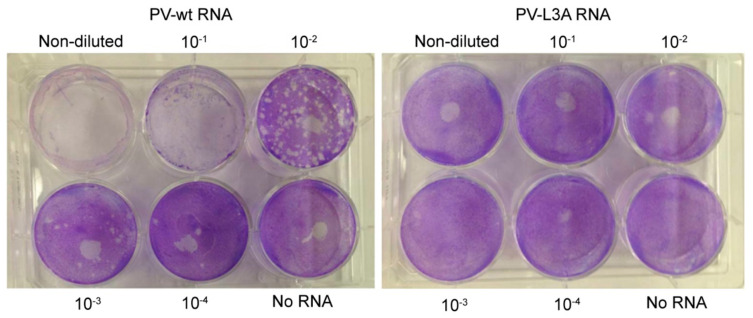
Mutations reducing helicity of the amphipathic α-helix of 2C protein block poliovirus infection. HeLa cells were transfected with serial dilutions of PV-wt or PV-L3A full-length poliovirus RNA transcripts. Cells were overlaid with DMEM growth medium containing FBS solidified with agarose at six hours post-transfection. To visualize plaques, the monolayer was stained with crystal violet at two days post RNA transfection.

Given our results that the L3A peptide was defective to tubulate liposomes, we expected that the mutations, when incorporated into the viral genome, would affect the viral replication. To test this, we introduced the specific mutations L3A to the full-length cDNA of PV-wt to construct the PV-L3A mutant. Transcripts of PV-wt and the PV-L3A mutant were subsequently produced and used in the plaque assay to examine their infectivity. HeLa cell monolayers were transfected with a series of diluted PV transcripts. As shown in Figure 8, the PV-wt infectivity was readily detected at two days post RNA transfection, as plaques can be observed at all dilutions. However, the PV-L3A infectivity was non-detectable, as no visible plaque was formed even at the non-diluted condition. These results suggested that the three mutated amino acids are critical for PV infection, and the affected remodeling of liposomes by the amphipathic α-helix might account for the defective infection.

## 4. Discussion

Rearranging the host cellular membrane to form VRCs is a highly conserved feature among (+)RNA viruses. These viruses replicate in designated organelle membranes, where viral replication proteins, in the majority of cases, facilitate or induce membrane rearrangements. For the viral replication proteins that do not have a transmembrane domain, they may have one or several amphipathic α-helices, such as poliovirus 2C (aa 19-36) [17,18], BMV 1a (helix A: aa 392-407; helix B: aa 416-433) [32,33], Hepatitis 5A (aa 7-28) [34], CHIKV nsP1 (aa 244-263) [35], and Semliki Forest virus (SFV) nsP1 (aa 245-264) [36,37]. It has been generally accepted that such amphipathic α-helices serve as an anchor for viral replication proteins to be associated with membranes. We report here that the synthesized peptide of the amphipathic α-helix from poliovirus 2C protein binds to and tubulates liposomes, and the generated tubules are stable for more than six days (Figure 3, Figure 4, Figure 5). Our data thus provide evidence that the amphipathic α-helix in poliovirus 2C protein not only serves as an anchor for 2C’s membrane association, but also plays an active role in the remodeling of membranes.

It has been demonstrated that synthesized peptides of BMV 1a helix A and the helix in SFV nsP1 only form helical structure upon binding to membranes, and are necessary and/or sufficient to target proteins to membranes [32,36,37]. Similarly, a recent report showed that the peptide of CHIKV nsP1 amphipathic α-helix also forms a helical structure when it binds to negatively charged lipid vesicles [35]. Although the purified CHIKV nsP1 protein was able to induce membrane remodeling, the peptide representing the amphipathic α-helix failed to reorganize liposomes [35]. In poliovirus 2C protein, the N-terminal fragment with 77 amino acids or 39 amino acids has been shown to be sufficient for the association with membranes [19] or LDs [21], respectively. In addition, the amphipathic α-helix in poliovirus 2C is necessary for the association with LDs [21]. These results indicate the critical role of the amphipathic α-helix for membrane association of poliovirus 2C protein. Our results for the first time confirmed that this predicted amphipathic α-helix in poliovirus 2C protein behaves as a helix: the 2C helix peptide increased helical conformation when binding to the lipid membrane, and the mutations in the hydrophobic face of the amphipathic region largely abolished this enhancement (Figure 5). In addition, sole 2C helix peptide is sufficient to induce the remodeling of the membrane structure (Figure 2, Figure 4 and Figure 5), in contrast to CHIKV nsP1, of which the cytoplasmic domain of nsP1 is also required for membrane reorganization [35]. Many cellular proteins contain such amphipathic α-helices that are necessary for their membrane-associated functions [38]. Previous studies with endophilin A1 has shown that there could be multiple helices in a single protein that can mediate or regulate membrane interaction in a protein [39,40]. Altering the charge of the central helix of endophilin A1 changed the insertion depth of this helix that altered membrane remodeling [39]. Another example is of epsin 1, which is also known to bind membranes through an amphipathic α-helix [41]. Any mutation that affected the hydrophobic face of helix 0 modulated the membrane interaction of the protein [41].

Overall, our data show that the 2C peptide from the N-terminal amphipathic domain of poliovirus 2C protein has enough structural features to induce membrane remodeling, and the three amino acid residues that are part of the hydrophobic face of this amphipathic region could have a critical structural role for membrane interaction of this domain. Both electrostatic and hydrophobic interactions contribute to the membrane binding of 2C peptides, however, the contribution from electrostatic interactions is small (Figure 3). This is evident from only a marginal decrease in the helical content of 2C-wt in the presence of POPG vesicles compared to POPC/POPE lipid compositions. Furthermore, 2C-L3A displayed only a small increase in helical content in the presence of fully negatively charged phospholipid vesicles. The hydrophobic interaction, on the other hand, should have a major influence on the membrane interaction of the 2C peptide, as supported by the fact that mutations affecting the hydrophobic face of the 2C peptide strongly disrupt membrane interaction (Figure 3).

The data from the plaque forming assay further underscore the role of the three hydrophobic amino acids in viral replication. Although the three substitutions may affect the processing of the 2BC intermediate to release 2C protein or the 2C protein conformation to interact with other viral or host proteins, it is highly likely that the inability of the 2C-L3A protein to infect HeLa cells is due to the compromising of the membrane remodeling ability. In the absence of detailed structural data, it is difficult to speculate how the mutations might affect the positioning of the amphipathic α-helix on the membrane surface. Further investigations to unravel the membrane-bound structure of the 2C amphipathic α-helix could provide answers to such questions.

Our observation provides the opportunity to design or search for ligands that can bind to the 2C N-terminal amphipathic α-helix, thereby disrupting the assembly of viral replication complexes and viral replication. Further studies could reveal whether targeting amphipathic α-helices in viral proteins, such as polioviral 2C, BMV 1a, nsP1 of SFV, or CHIKV, could be an antiviral approach in both plants and animals.
